# Xenogeneic Transplantation Promoted Human Exosome Sequestration in Rat Specific Organs

**DOI:** 10.34172/apb.2024.022

**Published:** 2023-12-04

**Authors:** Halimeh Mobarak, Mahdi Mahdipour, Arshad Ghaffari-Nasab, Reza Rahbarghazi

**Affiliations:** ^1^Biotechnology Research Center, Tabriz University of Medical Sciences, Tabriz, Iran.; ^2^Department of Applied Cell Sciences, Faculty of Advanced Medical Sciences, Tabriz University of Medical Sciences, Tabriz, Iran.; ^3^Department of Reproductive Biology, Faculty of Advanced Medical Sciences, Tabriz University of Medical Sciences, Tabriz, Iran.; ^4^Drug Applied Research Center, Tabriz University of Medical Sciences, Tabriz, Iran.

**Keywords:** Human exosomes, Intravenous administration, Rat, Cellular uptake, Off-target sequestration

## Abstract

**Purpose:**

Here, we aimed to study the distribution pattern of normal and cancer xenogeneic exosomes (Exos) and possible interspecies reactions in a rat model.

**Methods:**

Exos were isolated from normal Human umbilical vein endothelial cells (HUVECs) and MDA-MB-231 breast cancer cells. Diameter size and zeta potential distribution were studied using dynamic light scattering (DLS). The morphology of isolated Exos was monitored by scanning electron microscopy (SEM) images. Using western blotting, protein levels of exosomal tetraspanins were detected. For the *in vivo* study, Dil-labeled normal and cancer Exos were injected into the tail vein (100 µg exosomal protein/rat) three times at 1-hour intervals. After 24 hours, rats were euthanized and the cellular uptake of Exos was monitored in different organs using immunofluorescence staining (IF).

**Results:**

The size distribution and mean zeta potential of HUVEC and MDA-MB-231 cells Exos were 80±29.94 and 64.77±25.49 nm, and −7.58 and −11.8 mV, respectively. Western blotting revealed CD9, CD81, and CD63 in normal and cancer Exos. The SEM images exhibited typical nano-sized round-shape Exo particles. IF staining indicated sequestration of administrated Exos in splenic tissue and lungs. The distribution of Exo in kidneys, aorta, and hepatic tissue was less. These features were more evident in the group that received cancer Exos. We found no obvious adverse effects in rats that received normal or cancer Exos.

**Conclusion:**

Normal and cancerous xenogeneic human Exos can be sequestrated prominently in splenic tissue and lungs. Novel delivery approaches and engineering tools are helpful in the target delivery of administrated Exos to the injured sites.

## Introduction

 In higher creatures, intercellular communication is orchestrated via juxtacrine interaction or shuttling of soluble bioactive chemicals between varied cell types.^1,2^ In this regard, almost all cell types can release heterogeneous extracellular vesicles (EVs) with the origin of endosomes [exosomes: Exos], and plasma membrane [microvesicles] into the extracellular space.^1,3^ Currently, Exos, nano-sized vesicles with an average range diameter of 30-150 nm are considered putative therapeutic candidates.^4^ Molecular investigations have revealed that EVs, especially Exos, harbor diverse signaling molecules with the potential to regulate horizontally the activity of target cells.^5^ Different ways are contributing to the uptake of Exos by the host cells. The first inward transport system is endocytic mechanisms such as lipid raft-mediated endocytosis, caveolin- and clathrin-based endocytosis, and micropinocytosis, which greatly promote the entry of Exos into the target cell cytosol. In addition to the direct membrane fusion, the direct interaction of Exo surface ligands with cell surface receptors can also help the uptake procedure.^6-8^

 In recent years, whole-cell- and EV (Exos)-based therapies have been used along with conventional modalities for the treatment of various complications and abnormalities.^9^ Compared to the whole cell therapy, Exos can in part, but not completely, circumvent the problems associated with crossing biological interfaces and allo-recognition rejection.^4^ The field of Exo-based drug delivery is at the center of attention for increasing targeting efficiency.^10^ For this purpose, researchers have purified Exos from diverse biofluids with healthy and cancerous origins, and culture media using different protocols. However, the cellular uptake and intracellular trafficking of autologous/allogeneic/xenogeneic Exos have not been described yet. The injection of allogeneic and xenogeneic Exos can stimulate antigen-presenting cells and allo-/xeno-reactive responses.^11^ The physicochemical properties and high-rate *in vivo* biodistribution can increase the likelihood of elimination by hepatic and splenic macrophages.^12^ It should not be forgotten that the low levels of recognition elements on circulating Exo surface and rapid cell entry can reduce the direct interaction of Exos with immune cells compared to allogeneic/xenogeneic cells.^12^ In modalities associated with Exo therapy, the delivery of active compounds to the injured site is the subject of debate. Upon intravenous injection, the uptake of circulating Exo via macrophages leads to the accumulation in non-specific sites and reduction of the engraftment success in the target tissues.^13^ Thus, conducting relevant studies using allogeneic and xenogeneic Exos is essential to monitor the absorption rate and delivery efficiency after intravenous transplantation.

**Figure 1:**
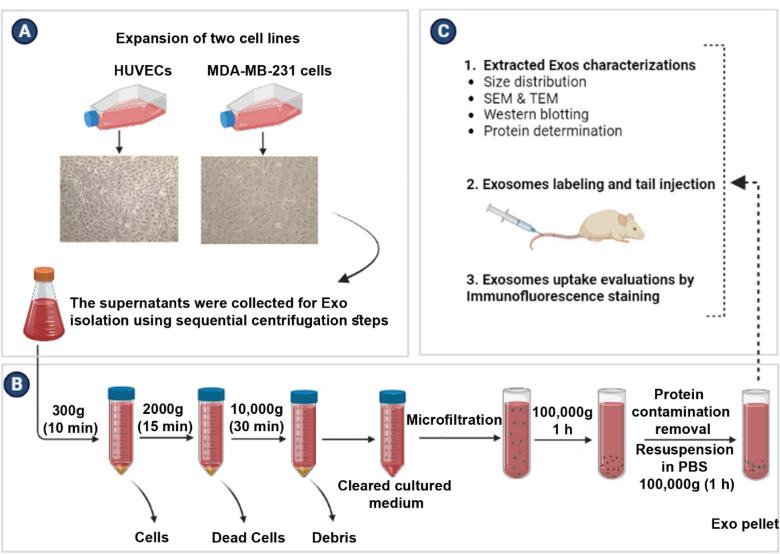
Experimental procedure flow chart (**A-C**). Normal Exos were isolated from human endothelial cell lines (HUVECs). For the isolation of cancer Exos, the human breast cancer MDA-MB-231 cell line was used (**A**). Isolation of Exos was done using the differential ultracentrifugation method (**B**). Characterization, and transplantation of normal and cancer Exos to a rat model (**C**)

 Here, we aimed to monitor the biodistribution pattern of xenogeneic Exos purified from human normal endothelial cells (ECs) and cancer breast cells in a rat model. It is hoped that the results of the current study can help us to understand the dynamic activity, biodistribution pattern of normal and cancer xenogeneic Exos, and possible reactions of immune organs after being administrated via the intravenous route (Figure 1A-C).

## Materials and Methods

###  Animal ethics 

 For experimental procedures, permission was obtained from the Local Committee of the Ethics at Tabriz University of Medical Sciences (IR.TBZMED.VCR.REC.1400.350).^14^ In this study, 15 male Wistar rats, ranging from 6 to 8 weeks old and weighing about 120 g, were used. Before starting the experiments, rats were acclimated for two weeks under standard conditions with free access to water and chewing food.

###  In vitro cell culture 

 To assess the Exo biodistribution pattern in rat xeno-transplant model, human normal and cancer Exos were isolated *in vitro* after the culture of human umbilical vein endothelial cells (HUVECs) and breast cancer MDA-MB-231 cells, respectively. Cells were purchased from Pasteur Institute (Iran) and expanded according to the previously described protocols.^15,16^ In short, cells were cultured using high-glucose content DMEM (DMEM/HG; Gibco) with 10% FBS (Gibco) and 1% Pen-Strep antibiotic solution (BioIdea Co.; Iran). Culture flasks were kept inside a CO_2_ incubator at 37 °C with a relative humidity of 95%. Upon reaching 70–90% confluency (Figure 1A), normal and cancer cells were passaged using Trypsin-EDTA solution (BioIdea Co.; Iran). In the current experiment, HUVECs and MDA-MB-231 cells at passages 3 to 6 were applied for several analyses.

###  Exosome purification 

 To isolate normal and cancer Exos, MDA-MB-231 cells, and HUVECs were cultured in the presence of Exo-free FBS for 48 hours. After that, supernatants were collected and Exos were enriched using the serial centrifugation method as previously described (Figure 1B).^16^ To exclude live and dead cells, samples were centrifuged at 300 g for 10 minutes and 2000 g for 15 minutes, respectively. Cell debris was also eliminated using centrifugation at 10 000 g for 30 minutes. After the completion of the centrifugation step, samples were micro-filtered. To obtain the Exo pellet, supernatants were centrifuged at 100 000 g for 60 minutes (Beckman Coulter Inc. Optima^TM^ TLX-120 ultracentrifuge). The procedure was continued by washing samples with phosphate-buffered saline (PBS) to remove any protein contamination and re-centrifugation at 100 000 gfor 60 minutes. All centrifuges runs were performed at a controlled temperature of 4 °C. Exo pellets were suspended in 200 μL PBS and stored at −80 °C for several analyses.

###  Measuring exosomal protein content

 To calculate the optimal Exo doses for transplantation, exosomal protein contents were measured (BCA Protein assay; Cat no: A101251; Protein Quantification Kit, Parstous Inc., Iran). Values were compared to the standard curve with R^2^ > 0.98 for each assay.

###  Exosome characterization 

####  Dynamic light scattering (DLS)

 In this study, the average size distribution and zeta (ζ)-potential values of isolated Exos were determined (Malvern Zetasizer Instruments, Herrenberg, Germany).

###  Morphological assessment 

 The morphology of normal and cancer Exos was investigated using scanning electron microscopy (SEM) microscopes as previously described.^16,17^ For TEM imaging, one drop (approximately 20 µL) of both purified Exos suspended in PBS was separately placed on carbon-coated 300-mesh copper grids and subjected to uranyl acetate staining (2% wt./v). Then, samples were covered with a carbon film. Electron micrographs were taken using a TEM at 100 kV (LEO 906, Zeiss, Germany). For SEM imaging, purified Exos were fixed in 2.5% PFA solution (Sigma–Aldrich), lyophilized, and gold-sputtered. Images were taken under an SEM instrument (Mira-3 FEG SEM microscope, Tescan Co., Czech).

###  Exosome immunophenotyping 

 Western blotting was performed to detect the Exo-related tetraspanins (CD9, CD63, and CD81). To this end, exosomal proteins were extracted using RIPA buffer and measured by BCA assay. Samples were electrophoresed on the 10% SDS-PAGE gel and transferred to PVDF membranes (Millipore). After blocking with 2% skim milk for 1 hour, membranes were incubated with primary anti-human CD9 (Cat no: sc-13118; Santa Cruz Biotechnology), anti-CD63 (Cat no: sc-5275; Santa Cruz Biotechnology), and anti-CD81 (Cat no: sc-166029; Santa Cruz Biotechnology) antibodies at 4°C overnight. The procedure was continued by several TBST washes (3 × 15 minutes) and incubation with HRP-conjugated secondary antibody for 1 hour. Immunoreactive bands appeared after incubation of membranes with a chemiluminescence kit (Cat no: ab65623; Abcam).

###  Exosomes labeling

 To track the Exo biodistribution in *in vivo* conditions, we used vital fluorescent dye. For this purpose, Exos were incubated with 20 µM Cell Tracker^TM^ CM-Dil dye (C5000; Invitrogen) for 20 minutes at 37 °C and washed with PBS before the transplantation.

###  Transplantation protocol

 Fifteen rats were categorized into 3 groups (n = 5) including Vehicle (rats that received only 10 µL sterile PBS); HUVEC derived-Exos (rats that received 100 µg exosomal protein in 10 μL PBS); and MDA-MB-231 cell derived-Exos (rats that received 100 µg exosomal protein in 10 μL PBS). The systemic injection was done via the tail vein three times with an interval of 1 hour. After 24 hours, rats were euthanized using an overdose of ketamine and xylazine. Tissues such as liver, lungs, kidneys, aorta, and spleen were sampled for subsequent analyses.

###  Immunofluorescence (IF) staining 

 The possible accumulation and uptake rate of transplanted Exos were studied using IF staining. The selected tissues were embedded in an OCT compound and sectioned into 5 µm slides using cryo-sectioning apparatus (Leica). After several PBS washes, samples were counterstained with DAPI (Dilution: 1 µg/ml; Sigma-Aldrich) and examined using an Olympus BX50 microscope.

###  Statistical analysis

 In this study, GraphPad Prism 8.0.1 software was used for data analysis. Statistical differences were measured using the one-way ANOVA test and Tukey post hoc method. *P* < 0.05 was considered statistically significant.

## Results

###  Exosome characterization and immunophenotyping

**Figure 2:**
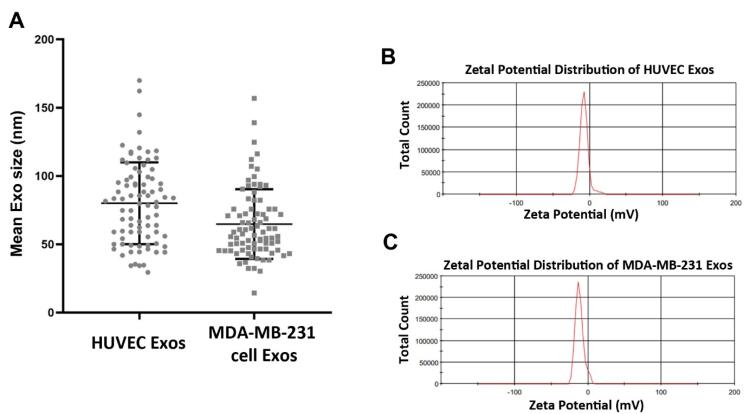
Particle size distribution of purified HUVECs-Exos and MDA-MB-231 cell-Exos, (**A**). Zeta potential distribution of isolated Exos analyzed by DLS (**B**)

**Figure 3:**
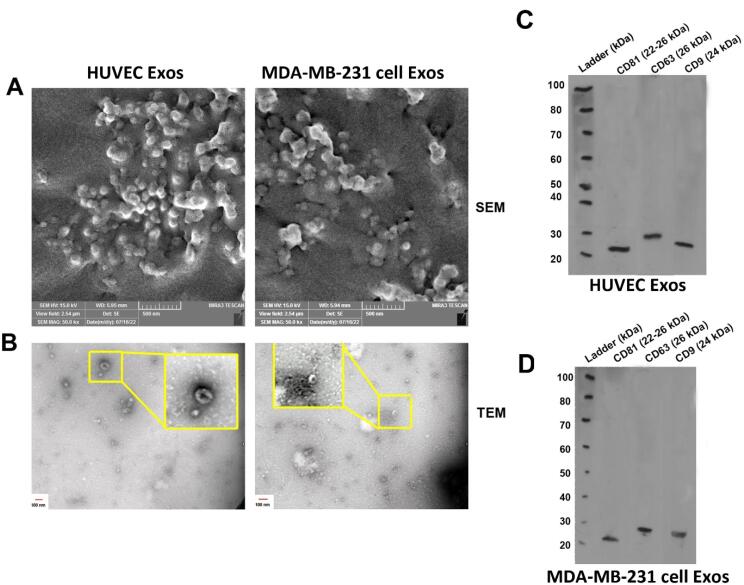
Ultrastructural analysis of normal and cancer Exos using SEM (**A**) and TEM (**B**) images. Data indicated spherical (**A**) and cub-shaped Exos (**B**) with multiple sizes. Western blot analysis was used to confirm the existence of tetraspanins (CD9, CD63, and CD81) in HUVEC-Exos (**C**) and MDA-MB-231 cell-Exos (**D**)

 In this study, the mean size distribution of the extracted Exos was determined using DLS. Data indicated the mean diameter size of 80 ± 29.94 and 64.77 ± 25.49 nm for Exos isolated from HUVECs and MDA-MB-231 cells, respectively (Figure 2A). According to our data, a mean zeta potential of −7.58 and −11.8 mV was obtained for purified Exos from HUVECs and MDA-MB-231 cells (Figure 2B-C). Exos were also visualized using SEM and TEM techniques (Figure 3A-B). Ultrastructural analysis SEM indicated spherical shape Exos with multiple dimensions in SEM images (Figure 3A). Agglomeration was evident in analyzed samples due to the drying process before imaging. According to the data, variation can be achieved in Exo size obtained from both cell types. Similarly, TEM images indicated negative shrunken particles with cup-shaped morphology that are identical to the Exos (Figure 3B). We noted the existence of surface tetraspanins CD9, CD81, and CD63 on isolated Exos from both cell lines (Figure 3C-D). Based on the data, Exos exhibited a relatively round shape appearance with no difference in the two groups. The morphological analysis confirmed that the size of cancer Exos was significantly smaller compared to normal counterparts.^18-20^

###  Uptake xenogeneic Exos in rat organs

**Figure 4:**
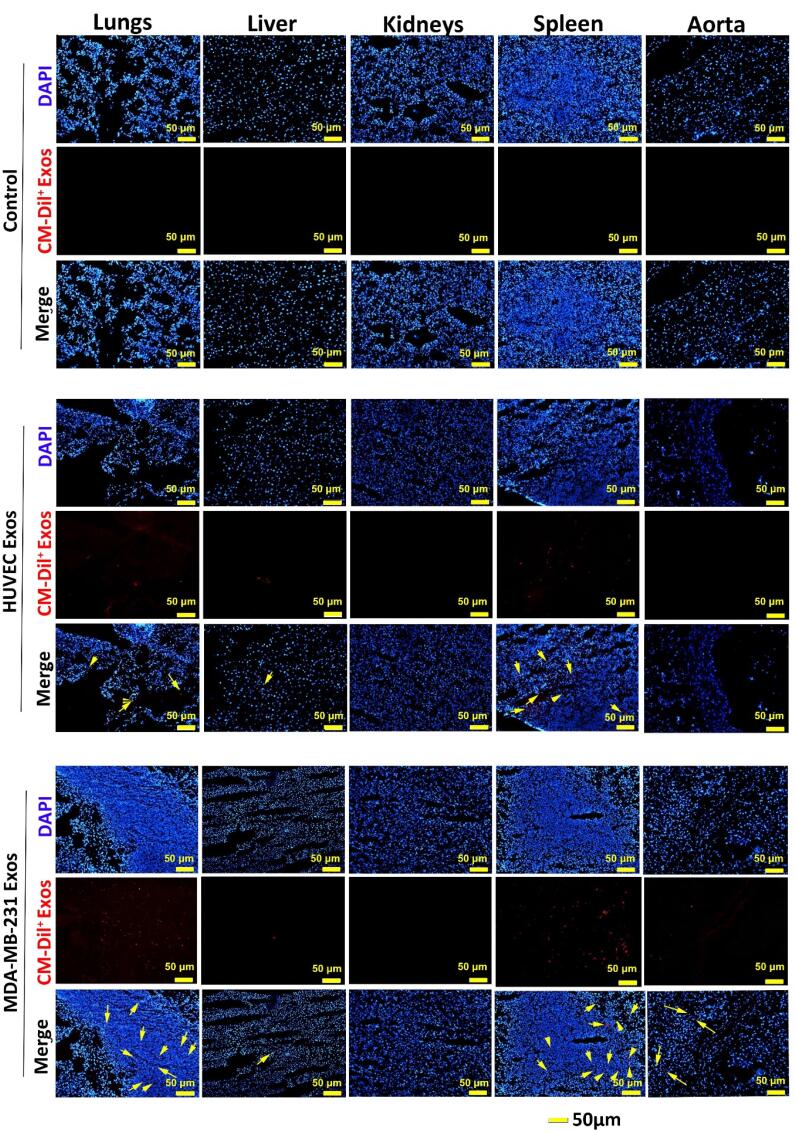
Immunofluorescence images of lungs, kidneys, liver, spleen, and aorta after intravenous administration of normal and cancer xenogeneic CM-Dil^+^ Exos in a model of rat after 24 hours. Data indicated that the lungs and spleen are the main tissue for intravenously administrated Exos and these values were more evident in rats that received cancer Exos (yellow arrows)

 Regardless of being isolated from cancer or a normal source, IF imaging revealed the internalization of both Exo types into cells in different tissue compared to the control vehicle group (Figure 4). In vehicle rats, no CM-Dil^+^ particles were detected in tissues such as the lungs, liver, kidneys, spleen, and aorta. We noted the existence of CM-Dil^+^ Exos in the spleen and lungs of rats that received normal HUVEC Exos. It seems that the intensity of recruited Exos into the splenic tissue was more compared to the pulmonary tissue. Partial fluorescence intensity was also detected in hepatic tissue without the sign of CM-Dil^+^ Exos in the aorta and kidneys (Figure 4). Data indicated the accumulation of cancer CM-Dil^+^ Exos in the pulmonary niche and with more intensity in splenic tissue. It seems that the number of cancer CM-Dil^+^ Exos was more in the lungs relative to the group that received normal CM-Dil^+^ Exos. Similarly, the intensity and number of cancer CM-Dil^+^ Exos were more in splenic tissue when compared to the rats that received normal CM-Dil^+^ Exos. We found no difference in the intensity and number of normal and cancer CM-Dil^+^ Exos in other tissue such as the liver, and kidneys (Figure 4). In contrast to normal Exos, the presence of cancer CM-Dil^+^ Exos was indicated in the aorta. These features showed that lymphoid tissues, especially splenic tissue, are the main target sites for transplanted xenogeneic Exos irrespective of purified from normal or cancer tissues. Due to the massive vascular network, Exos can be also directed toward the pulmonary tissue with less intensity than the spleen. It seems that the biodistribution of cancer Exos is high compared to the normal Exos, leading to insidious metastasis to varied tissue types.

## Discussion

 During recent decades, the application of allogeneic and xenogeneic Exos with cytoprotective properties has been rapidly increasing in various clinical trials.^4^ Xenogeneic Exos along with allogeneic Exos are touted as a valid therapeutic source for the alleviation of several pathologies.^21^ For example, Shi and colleagues indicated that the systemic administration of human Exos isolated can improve the healing of steatohepatitis in a mouse model.^21^ Notably, target delivery of Exos to the injured sites can contribute to the reduction of off-target therapeutic effects.^22^ Due to unique physicochemical properties, it has been shown that *in vivo* administered Exos easily distribute in biofluids and are cleared from circulation in a short time via the activity of phagocyte cells.^23^ Therefore, targeting Exos from different sources toward injured sites is considered to be the most favorable therapeutic approach.^24,25^ Although intravenous injection of Exos is a less invasive therapeutic approach it is not specified what fraction of total administrated Exos can reach the target sites. For all we know, there are few reports investigating the biodistribution of *in vivo* administrated xenogeneic Exos in animal models. This study was conducted to monitor the biodistribution of xenogeneic Exos from normal and cancer sources in a rat model. We also proposed that normal and cancer xenogeneic Exos may exhibit different biodistribution patterns.

 Here, we indicated that most fractions of injected Exos via intravenous approach were sequestrated in splenic tissues and pulmonary parenchyma. According to our data, the amount of recruited Exos to the splenic tissue and pulmonary parenchyma was more in rats that received cancer Exos compared to that of the normal Exos. It is thought that the existence of a bulk vascular network within the pulmonary niche is associated with the retention and quick trap of circulating Exos following intravenous injection.^4^ These microanatomical structures increase the possibility of reciprocal interaction of Exos with the luminal surface of ECs, leading to off-target therapeutic outcomes.^12,26^ In an experiment, it was shown that the Exo adherence property is associated with the degree of anaplastic change.^27^ Conigliaro and co-workers indicated that CD90^+^ hepatoma cells produce Exos with the capacity to alter the interaction of ECs with other cells by the regulation of ICAM-1.^27^ To be specific, these Exos can affect EC-to-EC juxtaposed interaction and permeabilize vascular interface. It seems that the type of cancer can lead to the production of Exos with specified surface markers like integrins.^28^ The increase of αvβ5 integrin on the exosomal surface can contribute to hepatic tissue accumulation via the direct interaction with Kupffer cells while other integrin types such as α6β4 and α6β1 increase the possibility of Exo direction toward pulmonary niche by lung fibroblasts and epithelial cells.^28^ Some authorities named the Exos as mini-cell units with the potential to carry specified parent cell contents to the target cells.^29^ It is believed that the existence of xeno-reactive peptide–MHC complexes on administrated Exos can result in the detection and retention by resident immune cells such as splenic dendritic cells, resulting in the activity of CD4^+^ and CD8^+^ lymphocytes.^30^ Besides, cancer cell Exos can transfer tumor-associated antigens with the potential to alter the surrounding microenvironment, leading to the activation of dendritic cells, NK cells, T lymphocytes, and macrophages.^31^ The direct exposure of dendritic cells to cancer cell Exos increases the expression of certain molecules such as CD80, CD86, and MHC-II, and stimulation of T lymphocytes.^32^ So it will not be surprising to say that the local accumulation of cancer cell Exos is higher compared to normal cell Exos in the reticuloendothelial system. Thus, the off-target effects of cancer cell Exos are more prominent as indicated in this study by IF staining. Despite these features, it is suggested that the promotion of allo- and xeno-reactive response is less after the injection of allogeneic and xenogeneic Exos in light of low levels of exosomal recognition elements like MHC-1 when compared to cellular counterparts.^12^ Likewise, the existence of anti-inflammatory factors like IL-10 and TGF-β can also diminish the activity of phagocyte cells.^11^ In this study, we did not observe adverse clinical outcomes in the rats that received normal or cancer xenogeneic Exos. It should not be forgotten that repeated doses of xenogeneic Exos can reduce cross-species tolerance and activate privileged immune cells.^12^ In an experiment conducted by Munagala et al, they indicated cross-species tolerance for milk Exos without the promotion of a pro-inflammatory response.^33^ The intravenous injection or oral ingestion of Dil^+^ Exos in mice led to maximum fluorescence intensity at early 24 hours. Based on the data, Exos are sequestrated in lungs, hepatic, pancreatic, and splenic tissues, kidneys, ovaries, colon, and brain in both administration routes.^33^ They indicated that Exos distribution is predominated in hepatic tissue while in our study lungs and splenic tissue are the main accumulation sites of administrated Exos. Like autologous Exos, several similar mechanisms such as endocytosis, surface protein-ligand interactions, direct cell membrane fusion, and micropinocytosis are involved in the entry of allogeneic and/or xenogeneic Exos to the cells.^3,34^ Among them, it is believed that cell surface membrane fusion is the main entry mechanism for Exo.^35-37^ Dong and colleagues found a lack of significant difference in the entry of allogeneic rat EVs and xenogeneic porcine EVs by rat adipose mesenchymal stem cells.^35^

## Conclusion

 The current study indicated that intravenously administrated xenogeneic human Exos are capable of entering into rat cells in several tissues. Both normal and cancer xenogeneic Exos are sequestrated in varied organs 24 hours after systemic injection. According to our findings, lungs and splenic tissue are the main sites for the accumulation of xenogeneic Exos. In modalities associated with systemic injection of xenogeneic Exos, lymphoid organs and activity of phagocyte cells should be prioritized for evaluation of therapeutic outcome and calculation of precise injection doses. Due to the low immunogenicity rate and lack of obvious clinical outcomes, it is suggested that xenogeneic Exos can be used in several animal models and possibly in clinical trials.^38^ Regarding the fact that undesirable side effects are less in normal xenogeneic Exos compared to the normal counterparts, thus the application of xenogeneic Exos from normal parent cells seems logical with the less unwanted outcome. To achieve more therapeutic outcomes, the development, and production of engineered Exos should be at the center of attention.

## Acknowledgments

 The authors would like to thank the personnel of the Faculty of Advanced Medical Sciences, Stem Cell Research Center, and Drug Applied Research Center, Tabriz University of Medical Sciences, Tabriz, Iran for guidance and help. This study was supported by a grant (68246) from Tabriz University of Medical Sciences.

## Competing Interests

 The authors declare that they have no competing interests.

## Ethical Approval

 All experimental procedures and protocols were approved by the Ethical Committee of Tabriz University of Medical Sciences (ethical code: IR.TBZMED.VCR.REC.1400.350) and conducted according to the Guidelines for the Care and Use of Laboratory Animals (NIH, 1986).
